# Harnessing probiotic-metformin synergy: targeting the gut-microbiota metabolism axis to ameliorate polycystic ovary syndrome

**DOI:** 10.3389/fnut.2025.1699600

**Published:** 2026-01-15

**Authors:** Wanqiu Yang, Lin Yin, Qingling Xie, Moyao Tan, Yunzhu Liu, Jili Xu, Jie Wu

**Affiliations:** 1 Hospital of Chengdu University of Traditional Chinese Medicine, Chengdu, Sichuan, China; 2 Yibin Second Hospital of Traditional Chinese Medicine, Yibin, Sichuan, China; 3 Hospital of Chengdu University of Traditional Chinese Medicine, Chengdu, Sichuan, China

**Keywords:** polycystic ovary syndrome, metformin, probiotics, gut microbiota, insulin resistance, AMPK, short-chain fatty acids

## Abstract

**Background:**

Polycystic ovary syndrome (PCOS) and type 2 diabetes mellitus (T2DM) are interrelated through insulin resistance (IR) and gut microbiota dysbiosis. This review evaluates the synergistic efficacy and mechanisms of a combined probiotic and metformin regimen in the management of PCOS, with a specific focus on the gut-ovary-metabolism axis.

**Methods:**

A systematic literature search was conducted following PROSPERO (ID: CRD420251143914) registration across PubMed, EMBASE, Web of Science, the China National Knowledge Infrastructure (CNKI), the Wanfang Database, and the VIP Database, prioritizing randomized controlled trials and retrospective studies.

**Results:**

The combination therapy suggests improved outcomes compared to metformin monotherapy, yielding significant improvements in metabolic, hormonal, and reproductive function. Crucially, probiotics alleviated metformin-induced gastrointestinal adverse effects, thereby enhancing treatment adherence. The proposed underlying mechanisms involve a synergistic restoration of gut microbial homeostasis, increased production of short-chain fatty acids, reinforcement of the intestinal barrier, and potentiated activation of the AMP-Activated Protein Kinase (AMPK) pathway.

**Conclusion:**

As an adjunct supplement to metformin, probiotics represent a novel, evidence-supported therapeutic strategy for PCOS. This approach offers enhanced efficacy and tolerability, with potential implications for a broader spectrum of metabolic disorders including T2DM; however, given the current paucity of related studies, conclusions should be drawn cautiously.

**Systematic review registration:**

[https://www.crd.york.ac.uk/prospero/], identifier [CRD420251143914].

## 1 Introduction

### 1.1 PCOS: a multifaceted endocrine disorder

Polycystic ovary syndrome (PCOS) is a common and heterogeneous endocrine–metabolic disorder affecting 5%–25% of reproductive-aged women worldwide (1). According to the 2023 International Evidence-based Guideline, diagnosis is based on the Rotterdam criteria, requiring at least two of the following: oligo-/anovulation, hyperandrogenism, or polycystic ovarian morphology (2). Beyond reproductive manifestations, PCOS is strongly linked to metabolic dysfunction, with insulin resistance (IR) present in 50%–70% of affected women and serving as a major driver of hyperandrogenism and future T2DM risk. Emerging systems-biology and microbiome evidence further suggests that chronic metabolic inflammation and gut dysbiosis act as upstream contributors to IR and hyperandrogenism in PCOS (3, 4).

### 1.2 The vicious cycle of insulin resistance and hyperandrogenism

Building upon the interconnected roles of IR and gut dysbiosis in PCOS, the relationship between IR and hyperandrogenism is not merely associative but constitutes a self-perpetuating “vicious cycle” that underlies the syndrome’s progression (5–7). IR in peripheral tissues induces compensatory hyperinsulinemia, which directly stimulates ovarian theca cells to overproduce androgens (8), while suppressing hepatic synthesis of sex hormone-binding globulin (SHBG) (9). The combined effect—enhanced androgen output and reduced SHBG availability—leads to elevated biologically active free androgen levels (10, 11). These excess androgens further impair insulin signaling in muscle and adipose tissue, thereby reinforcing IR and completing a detrimental feedback loop that amplifies both metabolic and reproductive abnormalities in PCOS (12). Understanding this bidirectional cycle positions IR as a central pathogenic driver and a primary therapeutic target.

### 1.3 The gut-ovary axis: a new frontier in PCOS pathophysiology

Emerging evidence has revolutionized our understanding of PCOS, highlighting the role of gut microbiota as a critical environmental factor that amplifies the IR-hyperandrogenism cycle (13–15). Women with PCOS consistently exhibit gut dysbiosis—an imbalance in microbial composition and function—characterized by reduced diversity and an altered ratio of key phyla (16–18). This dysbiosis contributes to increased intestinal permeability (often termed “leaky gut”), leading to the translocation of bacterial components like lipopolysaccharide (LPS) into circulation. The resulting “metabolic endotoxemia” triggers a state of chronic, low-grade systemic inflammation, which is a well-established driver of IR in both PCOS and Type 2 Diabetes Mellitus (T2DM) (10, 19–21). This “gut-ovary axis” provides a compelling rationale for interventions that target the gut microbiome to disrupt the core pathophysiology of PCOS.

### 1.4 Metformin and probiotics: two interventions converging on the gut

Metformin, an oral biguanide, remains the first-line pharmacologic therapy for managing metabolic disturbances in PCOS, particularly in patients with insulin resistance or obesity (22, 23). While traditionally thought to act systemically, recent evidence shows that metformin accumulates to high concentrations in the intestine, where it directly modulates the gut microbiota. This modulation is now considered a key part of its glucose-lowering mechanism (24, 25). However, these same gut-centric actions contribute to a high incidence of gastrointestinal (GI) side effects, which often limits treatment adherence (26, 27).

Probiotics, defined as live microorganisms conferring a health benefit, offer a direct approach to counteract dysbiosis and restore gut health (28, 29). They can improve intestinal barrier function, reduce inflammation, and produce beneficial metabolites like short-chain fatty acids (SCFAs) (30–32). Moreover, probiotics may mitigate metformin-induced gastrointestinal intolerance, supporting their use as both a stand-alone and synergistic therapy for PCOS (33, 34). Collectively, probiotic therapy provides a biologically grounded, natural product-based approach that complements pharmacologic modulation and forms the mechanistic basis for probiotic–metformin synergy.

### 1.5 Rationale and objectives of the review

Building on the mechanistic and therapeutic frameworks established in see sections “1.3 The gut-ovary axis: a new frontier in PCOS pathophysiology” and “1.4 Metformin and probiotics: two ointerventions cnverging on the gut,” the recognition of the gut–ovary axis and the established roles of both metformin and probiotics provide a compelling rationale for their combined use.

We hypothesize that this combination is synergistic, not merely additive: probiotics are postulated to not only directly ameliorate PCOS pathophysiology but also augment metformin’s efficacy by optimizing the gut environment and, crucially, mitigate its gastrointestinal adverse effects to improve adherence.

Based on this premise, this review aims to synthesize clinical and mechanistic evidence to elucidate this probiotic–metformin synergy. Specifically, we seek to:

- Decipher their interactive effects on shared signaling pathways, including AMPK activation, SCFA and bile-acid metabolism, and enteroendocrine signaling;
- Evaluate the combined impact on clinical hallmarks of PCOS, including insulin resistance, hyperandrogenism, and reproductive function; and
- Discuss the translational implications for developing personalized, microbiota-targeted therapeutic strategies.

By integrating recent meta-analyses and experimental studies, this review moves beyond summarizing efficacy to provide an updated, mechanism-based conceptual model that frames the gut microenvironment as a foundational therapeutic target and defines probiotic–metformin synergy as a novel paradigm in PCOS management.

## 2 Methods

This systematic review was designed and conducted following the PRISMA 2020 guidelines (visit^1^), and a completed PRISMA checklist is available in the Supplementary material. The review protocol was registered with PROSPERO (ID: CRD420251143914). The research question was structured according to the Population, Intervention, Comparison, Outcomes, and Study design (PICOS) framework.

### 2.1 Eligibility criteria (PICOS)

Population (P): Women of reproductive age diagnosed with PCOS according to the Rotterdam criteria or other internationally recognized standards, with or without associated metabolic conditions like insulin resistance, obesity, or T2DM.

Intervention (I): Co-administration of oral probiotics (any strain, single or multi-strain) and metformin.

Comparison (C): Metformin monotherapy or metformin with placebo.

Outcomes (O): Measures of glycemic control and insulin sensitivity (e.g., HOMA-IR, FPG, fasting insulin), and hormonal profiles (e.g., total testosterone, SHBG);Lipid profiles (e.g., HDL, LDL, triglycerides), reproductive function (menstrual regularity, ovulation rates), and incidence of gastrointestinal adverse effects.

Study Design (S): Randomized controlled trials (RCTs) and retrospective studies comparing the intervention and control groups were included.

### 2.2 Information sources and search strategy

A systematic literature search was conducted to identify all relevant studies published up to July 2025. The electronic databases PubMed, EMBASE, Web of Science, the Wanfang Database, and the VIP Database were comprehensively searched. The search strategy combined MeSH terms and keywords related to the population, intervention, and study design. The search was limited to studies published in English and Chinese. An example search strategy for PubMed is as follows: [(“Polycystic Ovary Syndrome” [MeSH Terms] OR “PCOS”) AND (“Metformin” [MeSH Terms] OR “Metformin”) AND (“Probiotics” [MeSH Terms] OR “Probiotics” OR “Synbiotics” OR “Gut Microbiota”)]

### 2.3 Study selection and data extraction

Two investigators independently screened titles and abstracts of the retrieved records against the predefined eligibility criteria. Full texts of potentially relevant articles were then obtained and assessed for final inclusion. Any discrepancies between the two investigators were resolved through discussion or consultation with a third reviewer. A standardized data extraction form was used to collect information on study characteristics (author, year), patient demographics, intervention details (probiotic strain, dose, duration), comparator, and outcomes (metabolic, hormonal, reproductive, and safety).

### 2.4 Risk of bias assessment and study quality

The methodological quality and risk of bias of the included studies were independently assessed by two reviewers. For randomized controlled trials (RCTs), the Cochrane Risk of Bias 2.0 (RoB 2.0) tool was applied, evaluating bias arising from the randomization process, deviations from intended interventions, missing outcome data, measurement of outcomes, and selection of the reported result. Each domain was rated as “low risk,” “some concerns,” or “high risk.” For the two non-randomized retrospective studies, the Risk of Bias in Non-randomized Studies of Interventions (ROBINS-I) tool was used, which examines bias due to confounding, participant selection, intervention classification, and selective reporting.

The overall risk-of-bias judgments are summarized in Table 1 and detailed in Supplementary Figure 1. Among the four RCTs, three were rated as having “some concerns” and one as “low risk,” primarily due to potential selective reporting and deviations from intended interventions, whereas randomization and handling of missing data were generally at low risk. Among the two retrospective studies, one showed “moderate” and one “serious” risk of bias, mainly driven by confounding and selection bias inherent to non-randomized designs.

**TABLE 1:** Risk of bias assessment of included studies.

References	Design	Randomization process	Deviations from intended interventions	Missing outcome data	Measurement of the outcome	Selection of the reported result	Overall risk
Xu et al. (37)	RCT	Low risk	Low risk	Low risk	Low risk	Some concerns	Some concerns
Chen et al. (38)	RCT	Low risk	Some concerns	Some concerns	Low risk	Low risk	Some concerns
Huang et al. (39)	RCT	Low risk	Some concerns	Low risk	Low risk	Low risk	Some concerns
Ji et al. (36)	RCT	Low risk	Low risk	Low risk	Low risk	Some concerns	Some concerns
Li et al. (40)	Retrospective	Not applicable (assessed with ROBINS-I: moderate risk)					Moderate risk*
Luo et al. (35)	Retrospective	Not applicable (assessed with ROBINS-I: serious risk)					Serious risk*

RCTs were assessed using the Cochrane RoB 2.0 tool. Non-randomized studies were assessed using the ROBINS-I tool; their inherent design limitations lead to a higher overall risk of bias (*).

Collectively, while the evidence indicates a consistent direction of benefit for the probiotic–metformin combination, the moderate-to-serious bias risk in several studies tempers the strength of conclusions. Hence, the findings should be interpreted with caution and regarded as hypothesis-generating. Future large-scale, high-quality RCTs with standardized protocols and transparent reporting are warranted to strengthen the evidence base.

### 2.5 Data synthesis

A narrative synthesis of the findings was conducted due to significant clinical and methodological heterogeneity across the included studies. A quantitative meta-analysis was deemed inappropriate because pooling data from such diverse sources would likely yield statistically and clinically meaningless results. The primary sources of heterogeneity included: (1) Intervention variability: Differences in probiotic strains (e.g., single-strain vs. multi-strain cocktails of *Bifidobacterium* and *Lactobacillus*), dosages, and treatment durations (12 weeks to 3 months); (2) Population differences: Variations in patient characteristics, such as obese versus non-obese PCOS phenotypes; (3) Study design: Inclusion of both RCTs and retrospective studies. Although a formal quantitative assessment of heterogeneity (e.g., using the I^2^ statistic or Cochran’s *Q*-test) was not performed as no meta-analysis was conducted, the clinical heterogeneity was judged to be substantial upon qualitative review. The findings are therefore presented thematically, structured according to the main outcome categories defined in the PICOS framework: metabolic, hormonal, reproductive, and safety outcomes. Subgroup analysis (e.g., based on PCOS phenotype or probiotic strain) and sensitivity analysis were planned but not executed due to the limited number of studies and the extensive heterogeneity, which would have rendered such analyses underpowered and inconclusive.

## 3 Results

### 3.1 Search results

**FIGURE 1:**
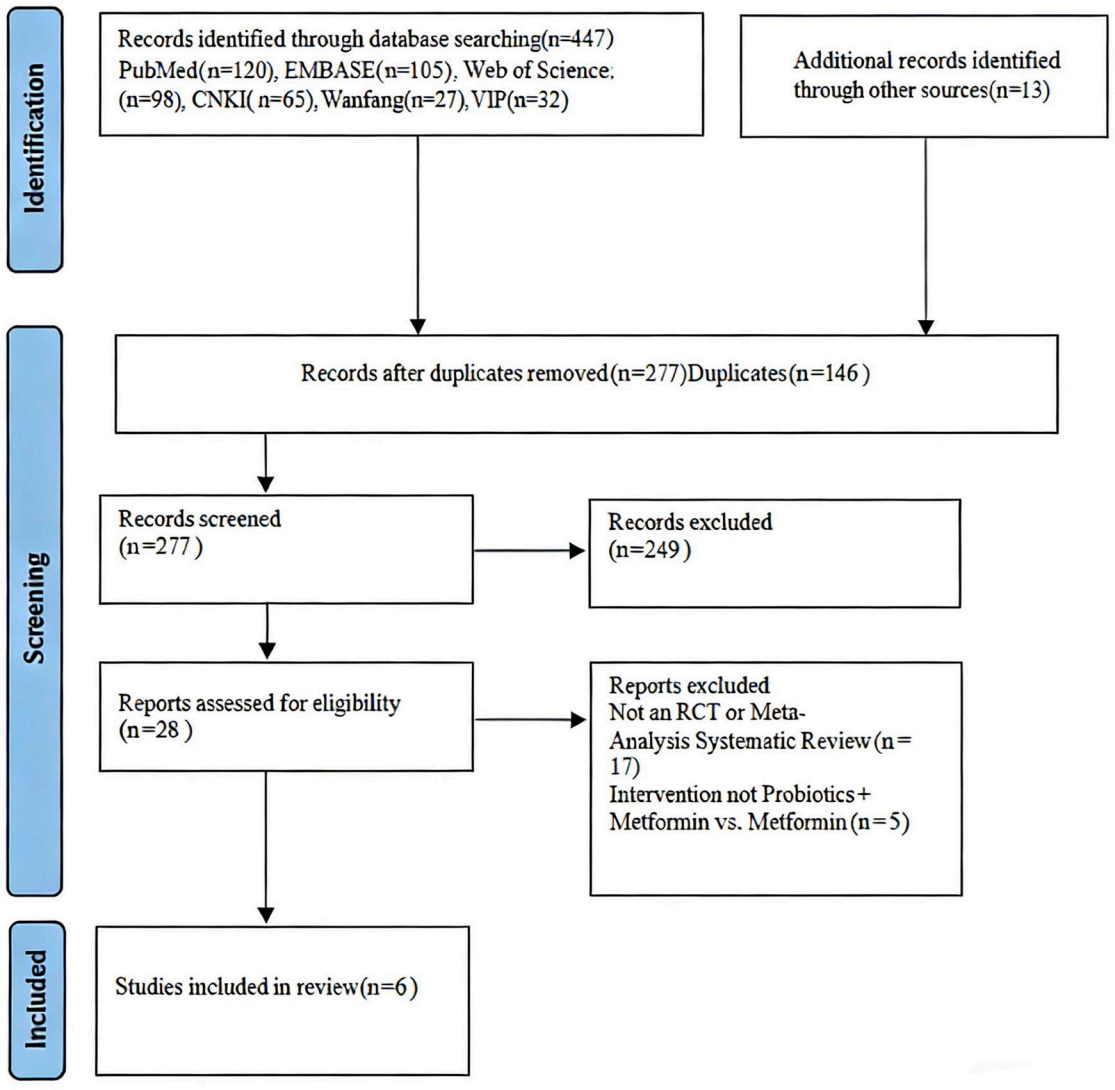
Preferred reporting ltems for systematic reviews and meta-analyses (PRISMA) diagram of study selection.

The study selection process is illustrated in Figure 1. A total of six studies met the inclusion criteria and were included in this systematic review. Characteristics of the included studies are summarized in Table 2.

**TABLE 2:** Characteristics and key findings of clinical studies investigating probiotic and metformin combination therapy in PCOS.

References	Design	Population	Intervention and duration	Positive findings	Contradictory/negative findings
Xu et al. (37)	RCT	80 PCOS women	3 months; metformin vs. metformin+*Bifidobacterium* quad-strain	Combination group had significantly lower FPG, FINS, HOMA-IR, TG, and VLDL-c.	No significant difference in TC, HDL-c, or LDL-c between groups.
Chen et al. (38)	RCT	55 PCOS patients (final N)	12 weeks; metformin vs. metformin+*Bifidobacterium* triple-strain	Combination group had significantly lower FBS, FSI, HOMA-IR, TG, VLDL-c, weight, and BMI. Also showed within-group reduction in LH and Testosterone.	No significant between-group difference in hormone levels (LH, Testosterone).
Huang et al. (39)	RCT	60 obese PCOS women	3 months; metformin vs. probiotics vs. combination	Combination group showed significantly greater reductions in weight, BMI, waist circumference, visceral fat, TG, LDL-C, and FBG, with higher HDL-C, compared to monotherapy groups.	No significant difference in anthropometric or metabolic improvements between metformin and probiotic monotherapy.
Ji et al. (36)	RCT	60 non-obese PCOS women	12 weeks; probiotics vs. metformin vs. combination	Combination group showed superior menstrual recovery (80%) and ovulation rates (75%) vs. monotherapy.	All groups showed similar improvements in metabolic markers (BMI, HOMA-IR, lipids).
Luo et al. (35)	Retrospective	141 obese PCOS women	3 months; metformin + diet vs. metformin + diet + probiotics	Combination group had significantly lower HOMA-IR, FPG, and androgen levels, with higher gut diversity.	No significant difference in post-treatment BMI between groups.
Li et al. (40)	Retrospective	138 PCOS patients	3 months; metformin vs. metformin + probiotics	Combination group showed significantly improved HDL, reduced insulin, and better anthropometric measures (BMI, waist).	Pilot study nature limits generalizability.

RCT, randomized controlled trial; PCOS, polycystic ovary syndrome; FPG, fasting plasma glucose; FINS, fasting insulin; HOMA-IR, Homeostatic Model Assessment for Insulin Resistance; TG, triglycerides; TC, total cholesterol; HDL-c, High-Density Lipoprotein Cholesterol; LDL-c, Low-Density Lipoprotein Cholesterol; VLDL-c, Very Low-Density Lipoprotein Cholesterol; BMI, body mass index; LH, luteinizing hormone; FBG, fasting blood glucose; FSI, fasting serum insulin.

### 3.2 Overview of clinical findings and heterogeneity

Evidence from four randomized controlled trials (RCTs) and two retrospective analyses supports the beneficial potential of combining probiotics with metformin in the management of PCOS. As detailed in Table 2, this combination has been evaluated across diverse PCOS phenotypes, demonstrating improvements in metabolic, hormonal, and reproductive outcomes. Notable heterogeneity was observed among studies in probiotic strain composition (*Bifidobacterium*, *Lactobacillus*), formulation (single- vs. multi-strain), dosage, and treatment duration (ranging from 12 weeks to 3 months), as well as in patient characteristics (e.g., obese vs. non-obese subtypes).

Overall, while study methodologies varied, the directional trends across outcomes remained consistent, indicating potential clinical synergy between probiotics and metformin.

### 3.3 Improvement in metabolic parameters

#### 3.3.1 Glycemic control and insulin sensitivity

Across included studies, combination therapy of probiotics with metformin showed a consistent trend toward improved glycemic control and insulin sensitivity relative to metformin alone. Luo et al. observed in 141 obese PCOS women that probiotic co-administration led to greater reductions in fasting plasma glucose (FPG) and HOMA-IR than metformin combined with diet alone (35). Ji et al. found comparable directional benefits in non-obese PCOS participants, with both metformin monotherapy and combination therapy improving glycemic markers, but the combination showing more pronounced effects (36). Xu et al., Chen et al. likewise reported lower FPG and HOMA-IR values in the combination group after 12 weeks to 3 months of treatment (37, 38). An RCT by Huang et al. on 60 obese PCOS patients also found that the combination group had significantly lower FBG levels compared to both metformin and probiotic monotherapy groups (39).

Collectively, these studies indicate a consistent direction of metabolic improvement, supporting an synergistic benefit of probiotics in enhancing metformin efficacy.

#### 3.3.2 Lipid profile

Dyslipidemia commonly accompanies PCOS-related insulin resistance. Several studies have shown that probiotics combined with metformin tend to improve lipid metabolism. Li et al. reported higher High-Density Lipoprotein Cholesterol (HDL) cholesterol and reduced fasting insulin and anthropometric indices with the combination regimen (40). Xu et al. (37) similarly observed lower triglycerides (TG) and Very Low-Density Lipoprotein Cholesterol (VLDL-c) levels in the combination group compared with metformin alone, whereas total cholesterol and Low-Density Lipoprotein Cholesterol (LDL-c) changes were less consistent across studies (37). Chen et al. (38) reported a downward trend in TG and VLDL-c with probiotic co-administration (41). The study by Huang et al. further reinforced these findings, showing that combination therapy led to significantly lower TG and LDL-C levels, and higher HDL-C levels, compared to either metformin or probiotics alone (39).

Overall, these findings suggest that probiotics may enhance metformin’s lipid-regulating effects, though inter-study variability limits direct comparison.

### 3.4 Regulation of hormonal and reproductive profiles

#### 3.4.1 Hyperandrogenism

Regarding hormonal outcomes, combination therapy appears more effective than metformin alone in reducing hyperandrogenemia. Luo et al. reported that patients receiving metformin and probiotics had significantly lower serum testosterone levels after treatment compared to those receiving metformin alone (35), indicating the greater improvements of combination therapy in reducing androgen levels. Similarly, a RCT involving adolescent PCOS patients revealed a greater reduction in testosterone levels and more significant improvement in acne severity scores in the combination group (26, 42). In the study by Chen et al. (38), while there was no significant difference between the combination and control groups post-treatment, the combination group did show a significant reduction in testosterone and luteinizing hormone (LH) levels compared to their own baseline (41).

#### 3.4.2 Menstrual cyclicity and ovulation

Clinical outcomes consistently indicated better menstrual regularity and ovulatory recovery with probiotic–metformin combination therapy. Ji et al. observed recovery rates of 40%, 55%, and 80% for probiotics, metformin, and combination groups, respectively (*p* = 0.035), with corresponding ovulation rates of 30%, 55%, and 75%, respectively (*p* = 0.017) (36). Other studies echoed this pattern, with improved reproductive function aligning with concurrent metabolic and hormonal improvement.

Overall, these findings suggest a synergistic trend toward restoration of ovulatory function when probiotics are co-administered with metformin.

### 3.5 Alleviation of metformin-induced side effects

A consistent and clinically meaningful observation across multiple studies is the improved gastrointestinal (GI) tolerability of metformin when combined with probiotics. A meta-analysis in patients with type 2 diabetes reported substantially fewer GI adverse events when probiotics were added to metformin therapy (43). Similarly, a clinical study demonstrated that multi-strain probiotics reduced the incidence of GI discomfort in patients previously intolerant to metformin (34). These results collectively highlight the potential of probiotics to mitigate metformin-induced GI side effects, potentially improving adherence and treatment continuity in PCOS management.

### 3.6 Summary of evidence

1. Across available clinical evidence, the co-administration of probiotics with metformin demonstrates a consistent trend toward improvements in glycemic control, lipid metabolism, androgen modulation, reproductive outcomes, and drug tolerability. However, the magnitude and statistical significance of these effects vary due to differences in study design, probiotic strain, dosage, duration, and PCOS phenotype. While the current evidence base remains limited, the overall direction of findings supports a biologically plausible synergistic effect, aligning with the gut–ovary–metabolic axis proposed in see section “1.3 The gut-ovary axis: a new frontier in PCOS pathophysiology.” These clinical findings lay the groundwork for mechanistic exploration, which is discussed in see section “4 Discussion.”

## 4 Discussion

### 4.1 Principal findings and clinical implications

The preliminary evidence supports that co-administration of probiotics with metformin offers substantial advantages over metformin monotherapy for women with PCOS. The synergy is evident across metabolic, hormonal, and reproductive domains, and notably, the combination improves metformin tolerability by reducing gastrointestinal (GI) distress. This dual action—enhancing efficacy while improving adherence—positions the combination as a rational therapeutic strategy. However, these conclusions should be interpreted cautiously due to small sample sizes and study heterogeneity. This consistent clinical pattern corroborates the gut–ovary–metabolic axis framework proposed earlier, linking clinical efficacy to an underlying biological plausibility.

### 4.2 Integrated mechanisms of metformin-probiotic synergy

**FIGURE 2:**
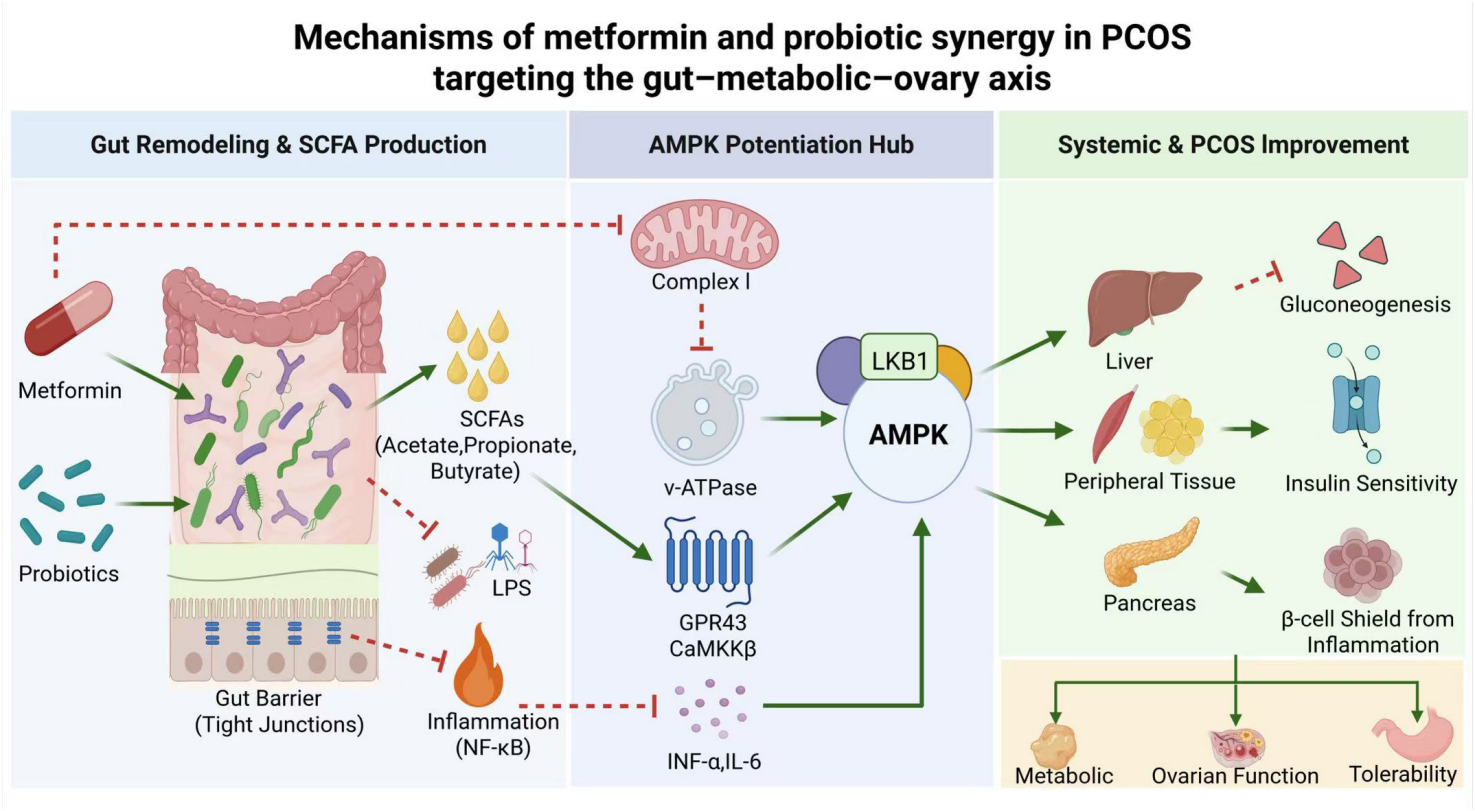
Mechanisms of metformin and probiotic synergy in polycystic ovary syndrome (PCOS) targeting the gut–metabolic–ovary axis. The schematic illustrates the proposed synergistic mechanisms. In the gut, metformin and probiotics act complementarily to enrich beneficial bacteria, increase short-chain fatty acid (SCFA) production, enhance barrier integrity, and reduce lipopolysaccharide (LPS)-triggered inflammation. These local changes converge to potentiate AMP-activated protein kinase (AMPK) activation via direct (metformin: Complex I inhibition, PEN2/v-ATPase/LKB1 axis) and indirect (probiotics: SCFA/GPR43 signaling, anti-inflammatory) pathways. Enhanced AMPK activity and improved gut–endocrine signaling [glucagon-like peptide-1 (GLP-1), peptide YY (PYY)] translate into systemic benefits: suppressed hepatic gluconeogenesis, increased insulin sensitivity in peripheral tissues, protected pancreatic β-cell function, and improved ovarian function, collectively disrupting the core pathophysiology of PCOS. (PCOS, polycystic ovary syndrome; SCFA, short-chain fatty acid; LPS, lipopolysaccharide; AMPK, AMP-activated protein kinase; GLP-1, glucagon-like peptide-1; PYY, peptide YY). Graphical conventions: Solid arrows (→) indicate activation; T-bar arrows (⟂) indicate inhibition.

The superior clinical outcomes observed with the combination therapy transcend a merely additive effect and originate from a multi-level, synergistic interplay between metformin and probiotics. This synergy is fundamentally rooted in their complementary actions on the gut ecosystem—remodeling the microbiome, modulating its metabolic output, and amplifying downstream host signaling pathways. Our proposed model for this integrated mechanism is illustrated in Figure 2.

#### 4.2.1 Dual modulation of the gut microbiome and its metabolites

The synergistic benefits of the combination therapy are rooted in its profound and complementary impact on the gut ecosystem. Metagenomic and metabolomic analyses reveal that the combined intervention is more effective at reshaping the gut microbial community than metformin alone.

Metformin’s role: Metformin significantly accumulates in the intestine—reaching concentrations 30–300 times higher than in plasma—and acts as a primary modulator of the gut ecosystem (24, 44). It alters microbial composition, for instance, by increasing the abundance of mucin-degrading *Akkermansia muciniphila* and several SCFA-producing bacteria. This enrichment is accompanied by increased circulating SCFAs (acetate, butyrate, propionate), which have been linked to improved insulin sensitivity, reduced fasting insulin, and modulation of host metabolic pathways (45–47). In addition, metformin-induced expansion of *A. muciniphila* correlates with enhanced intestinal mucin production and thickening of the mucus layer, which improves gut barrier integrity and reduces LPS translocation — thereby lowering systemic inflammation and contributing to metabolic benefits (48, 49).

Probiotic’s role: Probiotics — especially well-characterized SCFA-producing strains within the genera *Bifidobacterium* and *Lactobacillus* — can competitively inhibit pathogenic/pathobiont expansion, ferment dietary fibers to generate SCFAs and other beneficial metabolites, enhance tight-junction integrity, and reduce intestinal permeability(18, 28, 30). Through these actions they help to restore microbial balance, reduce endotoxin (LPS) load, and maintain an anti-inflammatory milieu in the gut (50, 51).

Synergistic impact: The combination leads to a more significant increase in microbial diversity and enriches beneficial genera more effectively than either agent alone (52). This results in an altered metabolic output, characterized by increased production of SCFAs and favorable modulation of bile acid profiles (53–55). These metabolites are key signaling molecules that improve insulin signaling, strengthen the gut barrier, and reduce systemic inflammation, directly aligning with the therapeutic goals of modulating gut-derived metabolites to restore systemic metabolic health (15). Collectively, this synergistically remodeled gut environment forms the mechanistic foundation for the downstream pathways discussed in the following sections.

#### 4.2.2 Potentiation of AMPK activation: a convergent metabolic hub

The synergistically remodeled gut milieu and its enriched output of SCFAs converge to potentiate a central regulator of energy metabolism: AMPK (56). Functioning as a metabolic master switch, AMPK orchestrates key processes including glucose uptake and lipid metabolism (57). The probiotic-metformin combination achieves more robust and sustained AMPK activation than either agent alone through complementary direct and indirect mechanisms.

Metformin activates AMPK via two primary pathways (25). First, through the canonical energy-sensing mechanism, it inhibits mitochondrial complex I, elevating the AMP/ATP ratio to promote allosteric activation and phosphorylation of AMPK (58–60). This mechanism has been extensively studied and experimentally supported. Research shows that upon activation, AMPK phosphorylates downstream substrates such as acetyl-CoA carboxylase (ACC), which inhibits fatty acid synthesis and promotes fatty acid oxidation, while also suppressing hepatic gluconeogenesis, thereby contributing to the reduction of blood glucose levels (60). Second, in addition to the classic energy-sensing pathway, metformin at therapeutic concentrations can also activate AMPK via an AMP-independent route (25). Specifically, metformin binds to the Presenilin Enhancer 2 (PEN2) subunit of the γ-secretase complex, recruits PEN2 to the lysosomal membrane, where it forms a complex with the ATPase H^+^ Transporting Accessory Protein 1 (ATP6AP1) subunit of vacuolar-type ATPase (v-ATPase), collectively inhibiting v-ATPase activity. Following the suppression of lysosomal acidification, the upstream kinase Liver Kinase B1 (LKB1) and the scaffold protein AXIN are relocalized to the lysosome, initiating the phosphorylation and activation of AMPK (61, 62). This latter pathway is particularly significant in high-drug-concentration tissues such as the intestine.

Probiotics indirectly prime the AMPK pathway by creating a favorable systemic milieu. First, probiotics ferment dietary fiber in the gut to produce SCFAs, such as acetate, propionate, and butyrate. These SCFAs not only serve as an energy source for intestinal epithelial cells but also act as signaling molecules to activate downstream pathways including AMPK via G protein-coupled receptors (GPCRs) (63). Studies have shown that propionate, a major SCFA, can activate AMPK by acting on the GPR43 receptor in peripheral tissues. For instance, upon binding to GPR43, propionate activates AMPK through Ca^2+^/calmodulin-dependent protein kinase kinase β (CaMKKβ), thereby inhibiting the expression of key gluconeogenic enzymes glucose-6-phosphatase (G6Pase) and phosphoenolpyruvate carboxykinase (PEPCK), which reduces hepatic glucose output (41). Similarly, SCFAs such as acetate can act on GPR41/43 receptors in adipose tissue and skeletal muscle, enhancing AMPK phosphorylation via CaMKKβ or other pathways, thus promoting lipolysis and glucose uptake (64). In summary, SCFAs produced by probiotics bind to GPR43 receptors in peripheral tissues and activate CaMKKβ, leading to the indirect activation of AMPK and improvement of systemic metabolic status (63, 65, 66). Second, probiotics can also enhance AMPK activity indirectly by improving intestinal barrier function and reducing inflammatory responses. Studies indicate that probiotics upregulate the expression of tight junction proteins (e.g., Occludin, Claudin, ZO-1), thereby strengthening the intestinal barrier and reducing permeability. This helps limit the entry of endotoxins such as LPS into the circulation and alleviates systemic inflammation. For example, inflammatory cytokines such as TNF-α and IL-6 may activate specific phosphatases or promote AMPK dephosphorylation, thereby impairing its function (67). AMPK itself also plays an important anti-inflammatory role by inhibiting the activity of the pro-inflammatory transcription factor NF-κB and reducing the production of inflammatory cytokines including IL-1β, IL-6, and TNF-α (68). Therefore, improving the gut barrier and lowering endotoxin and inflammation levels can release the “brake” on AMPK and restore its activity (68–70).

In summary, the combination therapy of probiotics and metformin synergistically activates AMPK through multiple layers and pathways. Metformin directly activates AMPK both via the classical energy-sensing mechanism and through the PEN2-mediated lysosomal pathway. Probiotics, on the other hand, activate AMPK in peripheral tissues through their metabolites, SCFAs, and further relieve the suppression of AMPK by improving the intestinal barrier and reducing systemic inflammation. This synergistic interaction leads to a more sustained and widespread enhancement of AMPK activity throughout the body, ultimately translating into significant improvements in downstream metabolic functions. These include enhanced insulin-mediated glucose uptake, more effective suppression of hepatic gluconeogenesis, and improved lipid metabolism homeostasis.

#### 4.2.3 Systemic signaling via the gut-metabolic-endocrine axis

In parallel to AMPK-mediated metabolic improvements, the restored gut environment also normalizes critical neuroendocrine communication along the gut-metabolic-endocrine axis. The gut communicates with distant organs, including the ovaries and brain, through neuroendocrine pathways. Microbial metabolites like SCFAs stimulate intestinal L-cells to secrete crucial gut hormones such as glucagon-like peptide-1 (GLP-1) and peptide YY (PYY) (55, 71, 72). These hormones act as key messengers linking intestinal microbial cues to peripheral organs including the pancreas, liver, adipose tissue, and reproductive system (72). In PCOS, disruptions in gut hormone secretion and SCFA availability impair ovarian steroidogenesis and follicular maturation, underscoring the importance of the gut–reproductive axis. In contrast, within T2DM, GLP-1 and PYY signaling predominantly affects β-cell insulin release and hepatic glucose output (20). This re-sensitized gut–metabolic–endocrine axis provides a mechanistic explanation for the superior improvements observed in systemic insulin regulation as well as reproductive outcomes—such as menstrual regularity and ovulation—in patients receiving combination therapy (73, 74).

### 4.3 Interpretation and integration with existing literature

The synergistic clinical outcomes observed go beyond additive effects, underscoring the systemic nature of PCOS pathophysiology centered on the gut–metabolic–ovary axis (12, 75). While the efficacy of metformin is traditionally ascribed to systemic AMPK activation, and that of probiotics to general gut health promotion, our integrated analysis leads us to propose that their confluence activates a virtuous cycle that disrupts the core pathophysiological loop of PCOS (18, 76). This model not only explains the superior clinical efficacy but also positions the gut microenvironment as a primary therapeutic target.

A pivotal insight from our mechanistic synthesis is the reconceptualization of AMPK not merely as a metformin target, but as a dynamic hub integrating signals from both host and microbiota. We delineate a dual-pathway model: metformin provides the direct pharmacological trigger (77, 78), while probiotics create a permissive metabolic milieu (e.g., through butyrate-induced energy provision and anti-inflammatory effects) (79, 80) that potently enhances cellular responsiveness to AMPK activation. This synergy likely underlies the enhanced insulin sensitization and may be particularly relevant in overcoming the AMPK suppression driven by chronic, low-grade inflammation in PCOS (57, 81, 82).

This dual-pathway interaction establishes a positive feedback loop: AMPK activation improves insulin sensitivity and reduces inflammation, which in turn supports the recovery of a beneficial microbial ecosystem. This reciprocal reinforcement aligns with the observed clinical synergy of probiotics and metformin, where improved gut homeostasis enhances pharmacological responsiveness and vice versa.

Furthermore, this integrative model explains the heterogeneity of anthropometric responses across clinical trials (83). The modest or inconsistent effects on BMI in some trials may reflect that the primary initial benefits of this combined gut-focused intervention are functional and metabolic (e.g., improved insulin signaling, hormone regulation) rather than structural (significant weight loss). This is consistent with studies in pre-diabetes, where metabolic improvement often precedes substantial weight reduction (83, 84). Consequently, the absence of dramatic weight loss should not be misconstrued as a lack of efficacy; rather, it highlights that the gut microbiome influences host physiology through multiple, weight-independent pathways (85, 86).

### 4.4 Strengths and limitations

The main strength of the current evidence lies in the consistency of directionality across heterogeneous studies. However, significant limitations persist, including variability in probiotic strains and dosages, small sample sizes, and short follow-up durations. Mechanistic evidence remains preliminary, with most studies lacking longitudinal microbiome or metabolomic profiling. Retrospective designs and moderate bias in some RCTs also temper confidence. Notwithstanding these constraints, the consistent, directional trend across methodologies supports the biological plausibility of this synergistic interaction.

### 4.5 Heterogeneity in probiotic strains and the challenge of strain specificity

A critical limitation, stemming from the heterogeneity mentioned above, is the lack of consistency in probiotic formulations. The interventions in the included studies ranged from a quad-strain Bifidobacterium preparation [Xu et al. (37)] and a triple-strain Bifidobacterium formula [Chen et al. (38)] (41) to other unspecified multi-strain cocktails (13, 35, 36). Such variability in strain composition, dosage, and duration complicates data interpretation and prevents direct comparison of efficacy across trials.

Probiotic effects are inherently strain-specific, as different microorganisms exhibit distinct metabolic and immunomodulatory functions. Therefore, it remains unclear whether the observed benefits result from a single potent species (e.g., *Bifidobacterium* lactis), a synergistic interaction among strains, or a general class effect. This ambiguity limits the formulation of evidence-based recommendations for clinical use.

This underscores the urgent need for future research to adopt standardized and clearly reported probiotic formulations. Head-to-head comparison trials of different strains and combinations are necessary to dissect the specific contributions of each and to identify optimal therapeutic regimens. From a clinical standpoint, while a definitive “best” probiotic cannot be identified from the current data, the consistent positive results from multi-strain preparations rich in *Bifidobacterium* and *Lactobacillus* suggest that such formulations are a rational choice for adjunctive therapy in PCOS management.

### 4.6 Future perspectives and translational potential

Despite these limitations, the findings have immediate clinical implications. For clinicians treating women with PCOS, considering the addition of a probiotic supplement to metformin therapy is a safe and evidence-supported strategy, particularly for improving metabolic parameters and treatment tolerability. The importance of strain specificity cannot be overstated; while evidence is still emerging, multi-strain formulations containing both *Lactobacillus* and *Bifidobacterium* species appear frequently in successful trials and represent a reasonable starting point.

The findings of this review extend beyond PCOS and offer significant insights for the management of T2DM. The ability of probiotics to mitigate metformin-induced GI distress can directly improve adherence in diabetic patients. The synergistic improvement in insulin sensitivity suggests potential for this combination in patients with an inadequate response to metformin monotherapy. Future research must prioritize large-scale, multi-center, long-term RCTs that employ standardized methodologies. These trials should directly compare different probiotic formulations to identify the most effective strains and dosages. Finally, incorporating multi-omics approaches (metagenomics, metabolomics) is essential to fully elucidate the synergistic mechanisms and identify biomarkers for personalized treatment strategies.

## 5 Conclusion

The addition of probiotics to standard metformin therapy represents a biologically plausible and promising strategy for the management of Polycystic Ovary Syndrome by synergistically targeting the gut–microbiota–metabolism axis. The current body of evidence indicates that this combination not only enhances the therapeutic efficacy of metformin in improving metabolic, hormonal, and reproductive outcomes but also improves treatment safety and adherence by mitigating gastrointestinal side effects.

This synergy appears to be driven by complementary actions of metformin and probiotics on gut microbial composition, SCFA and bile acid signaling, and AMPK-mediated metabolic regulation. However, these encouraging findings should be interpreted cautiously in light of the limited sample sizes, heterogeneity of probiotic formulations, and short study durations among available trials.

Future large-scale, rigorously designed randomized controlled trials with standardized probiotic strains and long-term follow-up are essential to confirm these benefits, refine dosage regimens, and establish this combined approach within clinical guidelines.

By elucidating how a natural microbial intervention can augment a first-line pharmacotherapy, this review provides a clinically and translationally relevant framework that extends beyond PCOS, offering new perspectives for microbiota-targeted strategies in metabolic and endocrine disorders.

## Data Availability

The original contributions presented in this study are included in this article/Supplementary material, further inquiries can be directed to the corresponding authors.

## Footnotes

1
https://creativecommons.org/licenses/by/4.0/
