# Supplementary material for: Harnessing probiotic-metformin synergy: targeting the gut-microbiota metabolism axis to ameliorate polycystic ovary syndrome
Source: Front Nutr. 2026 Jan 15;12:1699600. doi: 10.3389/fnut.2025.1699600 (PMC12854073; doi:10.3389/fnut.2025.1699600)
Supplement: Supplementary file 1 [file Supplementary_file_1.docx]

| **Section and Topic** | **Item #** | **Checklist item** | **Location where item is reported** |
| --- | --- | --- | --- |
| **TITLE** | | |  |
| Title | 1 | Identify the report as a systematic review. | Harnessing Probiotic-Metformin Synergy: Targeting the Gut-Microbiota Metabolism Axis to Ameliorate Polycystic Ovary Syndrome |
| **ABSTRACT** | | |  |
| Abstract | 2 | See the PRISMA 2020 for Abstracts checklist. | **Abstract** section provides a structured summary of the review's objective, methods, results, and conclusion. |
| **INTRODUCTION** | | |  |
| Rationale | 3 | Describe the rationale for the review in the context of existing knowledge. | **Introduction, sections 1.1 - 1.4**:：The rationale is built upon the limitations of metformin, the emerging role of the gut-ovary axis in PCOS, and the potential for synergistic therapy. |
| Objectives | 4 | Provide an explicit statement of the objective(s) or question(s) the review addresses. | Introduction, section 1.4 "Rationale and Objectives of the Review": "this review aims to synthesize evidence supporting the synergistic efficacy of probiotic-metformin co-therapy..." |
| **METHODS** | | |  |
| Eligibility criteria | 5 | Specify the inclusion and exclusion criteria for the review and how studies were grouped for the syntheses. | **Methods, section 2.1 "Eligibility Criteria (PICOS)"**: Population, Intervention, Comparison, Outcomes, and Study design are explicitly defined. |
| Information sources | 6 | Specify all databases, registers, websites, organisations, reference lists and other sources searched or consulted to identify studies. Specify the date when each source was last searched or consulted. | **Methods, section 2.2 "Information Sources and Search Strategy**"****: Databases (PubMed, EMBASE, Web of Science, CNKI) are listed. The search was up to **July 2025** (this date should be confirmed/updated to the actual search date). |
| Search strategy | 7 | Present the full search strategies for all databases, registers and websites, including any filters and limits used. | **Methods, section 2.2**: An example search strategy for PubMed is provided. The text states that the search was limited to English and Chinese. |
| Selection process | 8 | Specify the methods used to decide whether a study met the inclusion criteria of the review, including how many reviewers screened each record and each report retrieved, whether they worked independently, and if applicable, details of automation tools used in the process. | **Methods, section 2.3 "Study Selection and Data Extraction"**: "Two investigators independently screened titles and abstracts... Full texts... were then obtained and assessed for final inclusion. Any discrepancies... were resolved through discussion or consultation with a third reviewer." |
| Data collection process | 9 | Specify the methods used to collect data from reports, including how many reviewers collected data from each report, whether they worked independently, any processes for obtaining or confirming data from study investigators, and if applicable, details of automation tools used in the process. | Methods, section 2.3: Implied that the same two investigators used a "standardized data extraction form". It is recommended to explicitly state "Data were extracted independently by two reviewers" for clarity in the revision. |
| Data items | 10a | List and define all outcomes for which data were sought. Specify whether all results that were compatible with each outcome domain in each study were sought (e.g. for all measures, time points, analyses), and if not, the methods used to decide which results to collect. | **Methods, section 2.1 (PICOS - Outcomes)**: Lists metabolic, hormonal, reproductive, and safety outcomes. |
|  | 10b | List and define all other variables for which data were sought (e.g. participant and intervention characteristics, funding sources). Describe any assumptions made about any missing or unclear information. | **Methods, section 2.3**: "A standardized data extraction form was used to collect information on study characteristics (author, year), patient demographics, intervention details (probiotic strain, dose, duration), comparator, and outcomes..." |
| Study risk of bias assessment | 11 | Specify the methods used to assess risk of bias in the included studies, including details of the tool(s) used, how many reviewers assessed each study and whether they worked independently, and if applicable, details of automation tools used in the process. | Specify the methods used to assess risk of bias in the included studies, including details of the tool(s) used, how many reviewers assessed each study and whether they worked independently, and if applicable, details of automation tools used in the process. |
| Effect measures | 12 | Specify for each outcome the effect measure(s) (e.g. risk ratio, mean difference) used in the synthesis or presentation of results. | **Methods, section 2.5 "Data Synthesis**"**** (To be stated: Narrative synthesis; mean differences and percentages summarized) |
| Synthesis methods | 13a | Describe the processes used to decide which studies were eligible for each synthesis (e.g. tabulating the study intervention characteristics and comparing against the planned groups for each synthesis (item #5)). | **Methods, section 2.5** (All included studies were eligible for the narrative synthesis) |
|  | 13b | Describe any methods required to prepare the data for presentation or synthesis, such as handling of missing summary statistics, or data conversions. | Methods, section 2.5 (To be stated: Contacting authors planned for missing data; no conversions done) |
|  | 13c | Describe any methods used to tabulate or visually display results of individual studies and syntheses. | **Methods, section 2.5** (Results presented narratively and in tables/figures) |
|  | 13d | Describe any methods used to synthesize results and provide a rationale for the choice(s). If meta-analysis was performed, describe the model(s), method(s) to identify the presence and extent of statistical heterogeneity, and software package(s) used. | **Methods, section 2.5** (Rationale for narrative synthesis provided; meta-analysis deemed inappropriate) |
|  | 13e | Describe any methods used to explore possible causes of heterogeneity among study results (e.g. subgroup analysis, meta-regression). | **Methods, section 2.5** (To be stated: Not performed due to narrative synthesis) |
|  | 13f | Describe any sensitivity analyses conducted to assess robustness of the synthesized results. | **Methods, section 2.5** (To be stated: Not performed) |
| Reporting bias assessment | 14 | Describe any methods used to assess risk of bias due to missing results in a synthesis (arising from reporting biases). | **Methods, section **2.4**** (To be stated: Not performed due to small number of studies and narrative synthesis) |
| Certainty assessment | 15 | Describe any methods used to assess certainty (or confidence) in the body of evidence for an outcome. | **Methods, section 2.5** (To be stated: Not performed, e.g., GRADE) |
| **RESULTS** | | |  |
| Study selection | 16a | Describe the results of the search and selection process, from the number of records identified in the search to the number of studies included in the review, ideally using a flow diagram. | Results, section 3.1 "Search Results" and Figure 2 (PRISMA Flow Diagram) |
|  | 16b | Cite studies that might appear to meet the inclusion criteria, but which were excluded, and explain why they were excluded. | Figure 2 (PRISMA Flow Diagram) includes reasons for exclusion at the full-text stage. |
| Study characteristics | 17 | Cite each included study and present its characteristics. | **Results, section 3.1** and **Table 2 (Characteristics of included RCTs)** |
| Risk of bias in studies | 18 | Present assessments of risk of bias for each included study. | **Present assessments of risk of bias for each included study.** |
| Results of individual studies | 19 | For all outcomes, present, for each study: (a) summary statistics for each group (where appropriate) and (b) an effect estimate and its precision (e.g. confidence/credible interval), ideally using structured tables or plots. | Results, sections 3.4 - 3.6 (Narrative presentation with key statistics) and Table 2(Summarized data) |
| Results of syntheses | 20a | For each synthesis, briefly summarise the characteristics and risk of bias among contributing studies. | **Results, section 3.3 "Overview of Clinical Findings and Heterogeneity"** and **Table 1** |
|  | 20b | Present results of all statistical syntheses conducted. If meta-analysis was done, present for each the summary estimate and its precision (e.g. confidence/credible interval) and measures of statistical heterogeneity. If comparing groups, describe the direction of the effect. | **Results, sections 3.4 - 3.6** (Narrative synthesis results, describing direction of effect for combination therapy) |
|  | 20c | Present results of all investigations of possible causes of heterogeneity among study results. | Not performed. |
|  | 20d | Present results of all sensitivity analyses conducted to assess the robustness of the synthesized results. | Not performed. |
| Reporting biases | 21 | Present assessments of risk of bias due to missing results (arising from reporting biases) for each synthesis assessed. | Not performed. |
| Certainty of evidence | 22 | Present assessments of certainty (or confidence) in the body of evidence for each outcome assessed. | Not performed. |
| **DISCUSSION** | | |  |
| Discussion | 23a | Provide a general interpretation of the results in the context of other evidence. | **Discussion, sections 4.1 & 4.3** |
|  | 23b | Discuss any limitations of the evidence included in the review. | **Discussion, section 4.4 "Strengths and Limitations"** |
|  | 23c | Discuss any limitations of the review processes used. | **Discussion, section 4.4** (To be explicitly mentioned: Limitations of narrative synthesis and search strategy) |
|  | 23d | Discuss implications of the results for practice, policy, and future research. | **Discussion, section 4.5 "Future Perspectives and Translational Potential"** |
| **OTHER INFORMATION** | | |  |
| Registration and protocol | 24a | Provide registration information for the review, including register name and registration number, or state that the review was not registered. | **Methods, section 2"Registration" (PROSPERO 2025 CRD420251143914.)** |
|  | 24b | Indicate where the review protocol can be accessed, or state that a protocol was not prepared. | **Methods, section 2**(Implied by PROSPERO registration) |
|  | 24c | Describe and explain any amendments to information provided at registration or in the protocol. | Methods, section 2.4 (To be stated if any amendments occurred, or "No amendments were made.") |
| Support | 25 | Describe sources of financial or non-financial support for the review, and the role of the funders or sponsors in the review. | This research received no specific grant from any funding agency in the public, commercial, or not-for-profit sectors. |
| Competing interests | 26 | Declare any competing interests of review authors. | The authors declare that they have no known competing financial interests or personal relationships that could have appeared to influence the work reported in this paper. |
| Availability of data, code and other materials | 27 | Report which of the following are publicly available and where they can be found: template data collection forms; data extracted from included studies; data used for all analyses; analytic code; any other materials used in the review. | All data generated or analyzed during this study are included in this published article (and its supplementary information files). The full dataset and data extraction forms are available from the corresponding author on reasonable request. |

*From:*  Page MJ, McKenzie JE, Bossuyt PM, Boutron I, Hoffmann TC, Mulrow CD, et al. The PRISMA 2020 statement: an updated guideline for reporting systematic reviews. BMJ 2021;372:n71. doi: 10.1136/bmj.n71. This work is licensed under CC BY 4.0. To view a copy of this license, visit <https://creativecommons.org/licenses/by/4.0/>
