# Supplementary material for: Harnessing probiotic-metformin synergy: targeting the gut-microbiota metabolism axis to ameliorate polycystic ovary syndrome
Source: Front Nutr. 2026 Jan 15;12:1699600. doi: 10.3389/fnut.2025.1699600 (PMC12854073; doi:10.3389/fnut.2025.1699600)
Supplement: Supplementary file 2 [file Supplementary_file_2.docx]

****Figure S1. Summary of Risk of Bias Assessments for Included Studies.****


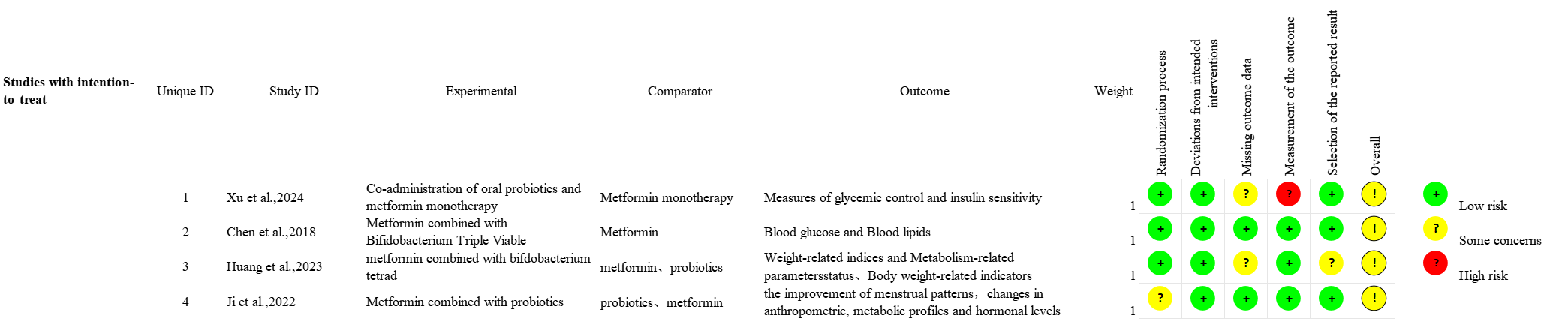


****(A)**** Risk of bias for randomized controlled trials (RCTs), assessed using the Cochrane RoB 2.0 tool. Each domain is judged as 'Low risk,' 'Some concerns,' or 'High risk.'


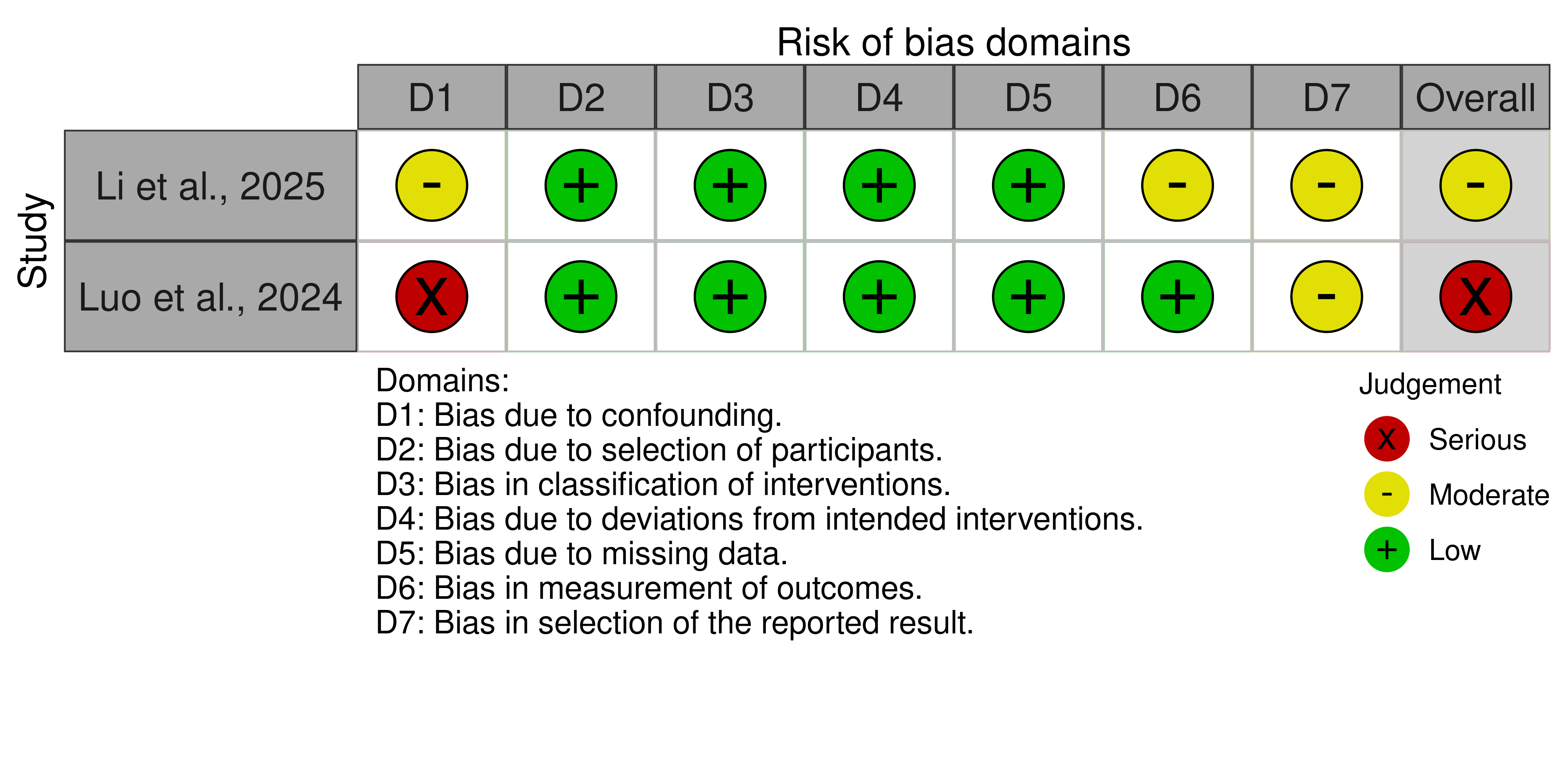


****
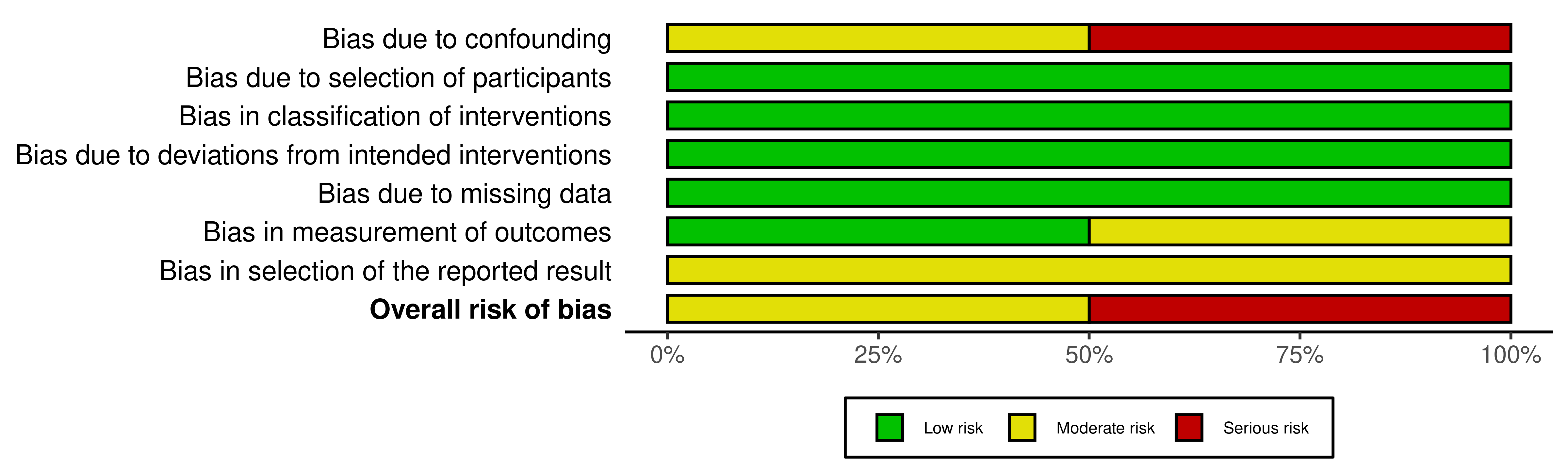
****

1. Risk of bias for non-randomized studies (retrospective studies), assessed using the ROBINS-I tool, and judged as 'Low,' 'Moderate,' 'Serious,' or 'Critical' risk of bias. The summary provides a visual overview of the proportion of studies with each judgment across the specified bias domains.
